# PReS-FINAL-2164: Agreement among musculoskeletal pediatric specialists in the assessment of radiographic joint damage in juvenile idiopathic arthritis

**DOI:** 10.1186/1546-0096-11-S2-P176

**Published:** 2013-12-05

**Authors:** R Pignataro, AL Rodriguez-Lozano, G Giancane, S Viola, M Valle, S Gregorio, X Norambuena, M Ioseliani, A Pistorio, A Martini, A Ravelli

**Affiliations:** 1 IRCCS Gaslini, Genova, Italy

## Introduction

The management of children juvenile idiopathic arthritis (JIA) is ideally conducted through the establishment of a multidisciplinary team of musculoskeletal pediatric specialists. Some therapeutic decisions, either medical or surgical, are made through discussion and consensus between specialists by viewing patient radiographs. However, it is unknown whether and to what extent different specialists agree in the assessment of the amount of radiographic joint damage.

## Objectives

The primary aim of the present study was to evaluate the agreement between musculoskeletal pediatric specialists in assessing structural joint changes in children with JIA.

## Methods

One pediatric rheumatologist, one pediatric radiologist and one pediatric orthopedic surgeon evaluated independently 60 radiographs of both wrists and hands, made in children with polyarticular-course JIA. Each specialist was asked to score each film using an adapted version of the Larsen score, whose score ranged from 0 to 5. Study radiographs were selected from 568 films used in a previous study aimed to validate an adapted pediatric version of the Sharp-van der Heijde (ash) score. To enable comparison of specialists' score with ash score, the 60 radiographs were divided in 6 classes of severity of damage, based on quintiles of ash score. Agreement was evaluated in terms of absolute agreement and through weighted kappa statistics.

## Results

The pediatric radiologist tended to assign lower scores and to provide more frequently 0 scores than did the other specialists. Absolute agreement ranged from 45% to 52%, depending on the pair of specialists examined. Both absolute and weighted kappa concordance between specialists' score and ash score were poorer for the pediatric radiologist than for the other specialists.

## Conclusion

We observed fair agreement in the assessment of radiographic damage among pediatric specialists involved in the care of children with JIA. The radiologist tended to be more reserved than the rheumatologist and the orthopedic surgeon in labeling radiographs as damaged or in considering changes as important.

## Disclosure of interest

None declared.

